# The Well-Forgotten Old: Platelet-Rich Plasma in Modern Anti-Aging Therapy

**DOI:** 10.3390/cells13211755

**Published:** 2024-10-23

**Authors:** Anna V. Gorodilova, Chulpan B. Kharisova, Maria N. Osinnikova, Kristina V. Kitaeva, Ivan Y. Filin, Yuriy P. Mayasin, Valeriya V. Solovyeva, Albert A. Rizvanov

**Affiliations:** 1 Institute of Fundamental Medicine and Biology, Kazan Federal University, 420008 Kazan, Russia; anagorodilova@yandex.ru (A.V.G.); harisovachulpan@gmail.com (C.B.K.); osinnikova.2003@gmail.com (M.N.O.); krvkitaeva@kpfu.ru (K.V.K.); ivyfilin@kpfu.ru (I.Y.F.); mayasin_yuriy@mail.ru (Y.P.M.); 2 Division of Medical and Biological Sciences, Tatarstan Academy of Sciences, 420008 Kazan, Russia

**Keywords:** platelet-rich plasma, platelet, regenerative medicine, aging

## Abstract

Currently, approaches to personalized medicine are actively developing. For example, the use of platelet-rich plasma (PRP) is actively growing every year. As a result of activation, platelets release a wide range of growth factors, cytokines, chemokines, and angiogenic factors, after which these molecules regulate chemotaxis, inflammation, and vasomotor function and play a crucial role in restoring the integrity of damaged vascular walls, angiogenesis, and tissue regeneration. Due to these characteristics, PRP has a wide potential in regenerative medicine and gerontology. PRP products are actively used not only in esthetic medicine but also to stimulate tissue regeneration and relieve chronic inflammation. PRP therapy has a number of advantages, but the controversial results of clinical studies, a lack of standardization of the sample preparation of the material, and insufficient objective data on the evaluation of efficacy do not allow us to unambiguously look at the use of PRP for therapeutic purposes. In this review, we will examine the current clinical efficacy of PRP-based products and analyze the contribution of PRP in the therapy of diseases associated with aging.

## 1. Introduction

The issue of preserving active longevity is a strategic challenge and a priority among scientists around the world. The pace of economic development and the availability of quality treatment have allowed us to increase life expectancy among both women and men, but diseases, many of which are chronic in nature, significantly reduce the quality of life of people from the onset of a certain age [1]. The direction that will be actively developed over the next decades is related to the search for mechanisms to improve the functioning of certain organ systems to keep people healthy and, consequently, maintain a longer working capacity for them.

Platelet-rich plasma (PRP) therapy was first introduced in the middle of the last century; by now, various platelet fractions have not only attracted the attention of specialists but have also entered widespread practice as a therapeutic approach to soft tissue repair, the healing of open wounds, the therapy of joint diseases, tendon and ligament repair, as well as solving esthetic problems [2]. First, the demand for these procedures is due to relatively labor-intensive sample preparation, the absence of serious reactions of the body during the introduction of autologous material, as well as the therapeutic effect achieved by realizing the natural functions of platelets. Under the general definition, PRP is the preparation of the autologous plasma of the patient, in which the concentration of platelets exceeds several times that of the initial level in the sample [3]. Once in the body, platelets included in PRP undergo activation that stimulates the secretion of various growth factors, such as platelet-derived growth factor (PDGF), transforming growth factor β (TGF-β), vascular endothelial growth factor (VEGF), epidermal growth factor, insulin-like growth factor (IGF), fibroblast growth factor (FGF), and many others [4]. Due to the extensive composition of bioactive components, PRP promotes tissue regeneration, wound healing, and the improvement of patients’ conditions [5,6,7]. While the number of clinical trials and registered PRP preparations is only increasing, there are several issues that require special attention and regulation: the introduction of a generally accepted classification of PRP, the standardization of sample preparation, and the need for platelet activation. In addition to the above-mentioned issues, in this review article we would like to draw attention to the effectiveness of PRP preparations in the treatment of diseases associated with aging, such as diseases of the musculoskeletal system, esthetic skin problems, and postoperative scars, as well as considerations of the prospects for the use of autologous plasma in the field of anti-aging therapy.

## 2. Senescence

The decline in infant mortality is attributable to the advent of modern medicines, vaccines, and medical equipment. The number of registered deaths among children under the age of five has decreased significantly since the year 2000, primarily due to a reduction in the number of deaths attributed to lower respiratory tract infections, diarrhea, complications of premature birth, intrapartum complications, malaria, and measles [1]. According to global statistics, life expectancy has increased from ~30 to ~73.5 years over the last century and a half [8]. This has resulted in a notable rise in the proportion of older individuals and an increase in the average age of the population in the majority of contemporary nations. In 1950, no country had a proportion of its population aged 65 and over exceeding 11%. In the year 2000, the highest figure was 18%. Nevertheless, the issue will intensify considerably by 2050, potentially reaching 38% [9]. Projections show that in 2050, there will be more seniors aged 60 and older than teens aged 10–24 years old [9]. However, today it is necessary to create conditions to prevent, slow down, and stop the processes of cellular aging, tissue reorganization, and organ dysfunction.

Cellular aging, first described in vitro in 1961, has become the focus of biotechnology companies seeking to improve various human conditions. Thus, Hayflick and Moorhead demonstrated in 1961 that normal cultured human fibroblasts exhibit a limited capacity for cell division before entering an irreversible growth arrest known as replicative senescence [10]. Aging is a process in which the physiological functions necessary for life deteriorate over time. The main factors leading to an increase in the number of senescent cells include the damage or shortening of telomeres [11], cellular metabolic changes [12], induction of p53 [13], epigenetic alterations [14], mitochondrial dysfunction [15], and reactive oxygen species accumulation [16].

The accumulation of senescent cells leads to chronic inflammation through the synthesis of pro-inflammatory cytokines such as interleukin (IL)-1β, IL-6, and chemokines IL-8 and C-C motif ligand 2, as well as several factors that determine the senescence-associated secretory phenotype of cells known as senescence-associated secretory phenotype (SASP) cells [17]. The SASP has a dual role in the cellular mechanisms of aging. Performing a protective function, several factors activate macrophages, natural killer cells and natural killer T cells, neutrophils, other cells of the immune system, and IgM, and ensure the elimination of old cells [18,19]. On the other hand, elevated levels of pro-inflammatory cytokines in the elderly lead to the development of chronic inflammation, this being a major endogenous risk factor for the development of age-related diseases: malignant neoplasms, cardiovascular pathologies, diabetes, and neurodegenerative diseases [20]. In addition, chronic inflammation accelerates the aging of immune cells, weakening the immune response, the inability to destroy senescent cells, and decreasing the antitumor activity of immune cells, which forms a vicious circle of inflammation and aging. As early as 1969, Walford proposed an “immunologic theory of aging”, which further developed into the concept of immunosenescence [21]. Thus, PRP therapy has the properties of modulating inflammation, reducing pain syndrome, and improving tissue regeneration.

## 3. Mechanism of PRP Work, Stages of Creation, Therapeutic Efficacy

Platelets are nucleus-free cellular elements differentiated from megakaryocytes by migration into the peripheral circulatory system, whose primary function is blood coagulation and thrombosis with subsequent arrest of bleeding [22]. However, platelets, like all blood-forming elements, perform a multifaceted range of functions, secreting a multitude of protein molecules, growth factors, and cytokines to improve tissue regeneration processes at the site of inflammation [23]. In addition, through the secretion of various cytokine molecules, platelets are able to attract immune cells to the focus of inflammation to activate the immune response [24]. It is this wide range of platelet functions and the ability to make a platelet-based product autologous (thereby not causing immune conflict) that led to the introduction of PRP into widespread practice. Now, more than 900 clinical trials have been registered on ClinicalTrials.gov, more than 40 PRP-based products are available on the pharmaceutical market for patients, and there is likely to be an increased trend of growth in the development and release of these drugs.

The general definition of PRP refers to autologous plasma in which, after a certain sample preparation in the blood, the concentration of platelets exceeds the original one by at least 2 times [6]. In this case, the question of how much the platelet concentration should exceed the physiological norm remains open, largely due to the lack of standardization of the methodology [25]. Since the use of plasma became popular, there have been many modifications of approaches, variations in protocols, and ways to evaluate the effectiveness of therapy. In this regard, several groups have developed systematics to identify a particular PRP product depending on the presence or absence of additional components (leukocytes, fibrin, various substrates). These modifications include platelet-rich fibrin, leukocyte-rich PRP, and leukocyte-poor PRP (LP-PRP) [26,27]. This article will focus on the role of classical PRP (by most classifications LP-PRP) in the therapy of diseases associated with aging, with a separate discussion on the need for plasma activation prior to direct application.

When platelets are activated, many biologically active molecules are released from their internal contents. It is this property of platelets that is decisive when PRP therapy is prescribed. Various growth factors and pro-inflammatory and anti-inflammatory molecules are located beneath the cytoplasmic membrane layer, mainly in alpha-granules and lysosomes, and it is these intracellular compartments that, when activated, release the molecules required for PRP. Mostly growth factors, cytokines, chemokines, immune mediators, and angiogenic factors are concentrated in the alpha-granules, while the delta-granules contain molecules necessary for blood coagulation [28]. Thus, according to sources, platelets may contain more than 600 bioactive components, which are in different concentrations and may even perform antagonistic functions in relation to each other [29]. Nevertheless, by concentrating platelets in plasma, we are able to obtain the necessary concentration of its therapeutic constituents.

The list of bioactive molecules released from platelets includes various growth factors, angiogenic factors, chemokines, immune proteins, and antibacterial compounds, so PRP locally can not only promote regeneration of the dermis and epidermis but also have a complex healing effect [30].

### 3.1. The Regenerative Potential of PRP

As an adjuvant therapy, PRP is successfully used for tissue regeneration. Here, it is worth noting the existence of a significant number of clinical cases of the healing of trophic venous leg ulcers, diabetic ulcers, and purulent lesions, and, of course, PRP is especially often used in esthetic medicine—in the treatment of alopecia, restoration of skin elasticity, etc. [31,32,33].

PDGF and TGF-β are involved in the synthesis of new collagen at the site of tissue damage. VEGF can stimulate the release of matrix metalloproteinases (MMPs), which play an important role in the remodeling of tissue components. IGF-1 and FGF factors act as signaling molecules for the proliferation and differentiation of mesenchymal stem cells (MSC); these factors also have a similar powerful effect not only on MSC but also on fibroblasts, chondrocytes, and osteocytes [34,35].

**Figure 1:**
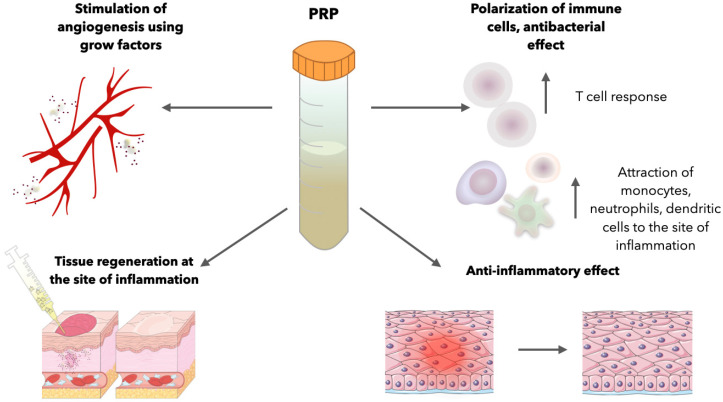
The main directions for using the therapeutic potential of PRP. Due to the presence of multiple growth factors and immunomodulatory agents and their release at the time of activation, PRP is able to relieve local inflammation in soft tissues, promote tissue regeneration in non-healing ulcers and wounds, stimulate angiogenesis, and direct various immune components to fight potential infection [25].

The successful regeneration of any tissue requires the formation of new blood vessels for tissue nutrition, the transportation of gasses, and other bioactive substances. This process is favorably influenced by VEGF, which is released during platelet activation. In addition, factors such as PDGF and TGF-β, which are also components of platelet granules, can stimulate VEGF secretion [36]. It has also been demonstrated in experiments that FGF also stimulates the process of angiogenesis [37,38] (Figure 1).

### 3.2. The Anti-Inflammatory Effects of PRP

Chronic inflammation is seen in many diseases, especially in the presence of non-healing diabetic ulcers, joint disease and entails pain syndrome, disease progression, and the risk of oncotransformation [39]. Many studies clearly show a trend of favorable effects of PRP injections at the site of inflammation, but few studies have investigated the molecular mechanisms and targets of PRP therapy. Thus, it has been realized that PRP therapy can reduce the protein expression levels of MMP13, A disintegrin, and metalloproteinase with thrombospondin motifs 5 and p65-transcription factors of the NF-kB signaling pathway, in a mouse model of knee osteoarthritis. In this case, PRP application led to a reduction in inflammation by decreasing the expression of the pro-inflammatory cytokines IL-1 and IL-6, and the tumor necrosis factor α (TNFα) [40]. The role of PRP in a mouse model of post-traumatic joint contracture was also demonstrated; the experiment concluded that PRP reduced the phosphorylation of signaling molecules of the TGF-β1/Smad signaling pathway and contributed to the inhibition of fibroblast-to-myofibroblast transformation, as well as reducing the expression of markers associated with fibrosis: alpha-smooth muscle actin, TGF-β, and collagen type I [41].

### 3.3. The Immunomodulatory Effect of PRP

At the site of inflammation, the immunomodulatory properties of PRP can lead to effective wound healing. Platelets at the site of inflammation help to maintain the necessary inflammatory environment and polarize immune cells into the desired phenotype, indirectly mediating protection against pathogens [42,43]. During activation, platelet granules have been shown to release a variety of antibacterial compounds active against both Gram-positive and Gram-negative bacteria. These compounds include peptide 3, chemokines, thymosin β-4, fibrinopeptide A, and fibrinopeptide B. These compounds can inhibit bacterial multiplication by making the membrane more permeable [44]. Platelets in PRP, with the help of surface immunomodulatory molecules such as *p*-selectin and Toll-like receptors, are able to initiate a cascade of immune reactions [45]. With the help of ligands on the platelet surface and the secretion of chemokines, they are able to attract monocytes, dendritic cells, and neutrophils to the site of inflammation and influence their differentiation [46,47]. In addition to interacting with innate immune cells, platelets indirectly stimulate the adaptive immune T/B-cell-mediated response, largely through CD40-CD40L receptor–ligand interactions [48].

### 3.4. Sample Preparation Procedures

In general, the sample preparation procedure is very simple and does not require expensive equipment. At the first stage, venous blood is collected, and here we can trace two directions: the use of commercial PRP preparation kits and the independent preparation of PRP. In the first option, manufacturers provide a wide range of techniques to obtain the final product. One of the main advantages of this method is the sterility of the PRP preparation, as, in most cases, such commercial kits imply a closed system where the intermediate and final products are not in any way in contact with the environment [49,50]. Table 1 shows a range of some of the commercial systems currently available on the market (Table 1).

However, studies that compare the quality characteristics of manufacturer-prepared kits and self-prepared PRP at the institution have increasingly emphasized the much lower platelet yield in commercial kits. For example, Gupta et al. in their paper compared the platelet yield of self-prepared PRP (using the two-step centrifugation method) and a commercial kit. Their study showed the concentration in homemade PRP was 7.25 ± 4.74 × 10^5^/µL, while when prepared by a commercial kit was 2.58 ± 0.81 × 10^5^/µL. However, this study compares only one parameter, the platelet count, and does not provide data on spontaneous premature activation and cytokine counts [51]. Ready-made kits, at first glance, may guarantee more reproducible results, but the initial biochemical parameters of patients and qualification of the specialist performing sample preparation do not make conclusions about the quality of the final procedure so unambiguous [52].

The open sample preparation procedure is a way of preparing PRP by a specialist without special kits. As a rule, in this case, the blood is centrifuged twice. The first centrifugation leaves a platelet fraction in the plasma supernatant, which in turn is confined from the erythrocyte sediment by a leukocyte film [53]. In the second step, platelets are concentrated from the supernatant, which end up in the precipitate. It should be noted that the number of revolutions and centrifugation time have not yet been definitively regulated. In any case, these various variables fall within the optimal range of 100–300 g (5–10 min) for the first centrifugation and 400–700 g (10–17 min) for the second centrifugation [54]. The main disadvantage of independent preparation is the possible risk of contamination of the product, so, in this case, aseptic measures should be properly observed to avoid side effects in the patient.

Although PRP has been shown to be not only safe but also effective in many areas, there are some difficulties, one of the most obvious being the non-reproducibility of the results. Non-reproducibility is due to the lack of clear regulations on sample preparation procedures, a comprehensive analysis of the results, and non-standardized variables—a non-fixed centrifugation speed, initially different levels of platelets in the patients’ blood, non-regulated platelet concentration in the final product, and the inhibitory effects of other blood components (e.g., red blood cells) [55]. Although serious side effects from the PRP procedure are extremely rare, the above details can ultimately lead to a complete lack of positive results, indicating the effectiveness of PRP therapy.

## 4. PRP Activation

Platelet activation is an important and integral step in the maintenance of normal hemostasis. During this process, platelets exhibit the ability to adhere to the site of vessel wall damage because of the mediated binding of subendothelial matrix proteins to glycoprotein receptors on the platelet surface [56]. Another subsequent step as a result of activation is platelet aggregation and white clot formation to stop bleeding, with the participation of multiple receptors and ligands such as von Willebrand factor, fibrinogen, and fibronectin [57]. Platelet activation results in a change in the disk-shape of the cell due to the formation of a large number of actin pseudopodia, thus forming aggregates with each other and other cells [58]. 

The platelet contains ~70 granules, which are categorized into three types: α-granules, dense granules, and lysosomal granules. In addition to membrane-binding receptors αIIbβ3 and *p*-selectin, α-granules also contain more than 300 soluble proteins involved in hemostasis maintenance (clotting factors), immunomodulatory functions (chemokines and cytokines such as CXCL1 and IL-8), and regenerative (VEGF, FGF) functions [59]. The exocytosis of α-granules is assessed by the expression of the CD62P marker, *p*-selectin, on the plasma membrane using flow cytofluorimetry [60], or by the release of granule contents such as von Willebrand factor [61]. Dense granules or δ-granules are less common platelet granules. They are 3–8 in number, while the number of α-granules is 50–80 per platelet. Dense granules contain small molecules (serotonin, histamine, ADP, ATP, calcium) [62]. The exocytosis of δ-granules can be assessed using lumi-aggregometry to detect released ATP [63]. There are few lysosomal granules and they contain hydrolases and degradative enzymes [64]. The exocytosis of lysosomal granule contents is usually assessed by evaluating the release of lysosomal enzymes such as beta-hexosaminidase [65].

Thus, platelet adhesion to subendothelial matrix proteins triggers a cascade of reactions to activate the cell. This key step leads to conformational changes, degranulation, and the release of the contents of cytoplasmic granules of platelets. At present, the question of the necessity of plasma activation remains open; some studies emphasize the activation stage, while in some works, the stages of exogenous activation are absent.

**Figure 2:**
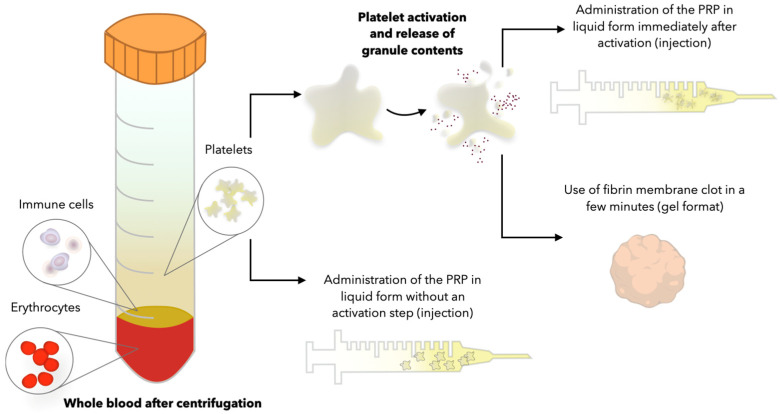
Application of different modifications of plasma platelet concentrates.

Although a greater effect is expected with activated platelets due to the higher content of growth factors, it is important to note that insoluble fibrin networks are also formed [66,67]. The fibrin network forms a three-dimensional framework that promotes cell migration and the retention of small molecules, but it can also serve as an obstacle when performing the injection [68]. To date, the results of research into the regenerative potential of PRP can be controversial due to different methods of PRP preparation, different concentrations of PRP, and the use of activated or inactivated PRP. In this section, the methods of PRP activation will be discussed, and the comparative characterization of the effect of activated and non-activated PRP will be presented (Figure 2). 

Physiologically, platelet activation occurs after contact with the injured blood vessel wall and subsequent interaction with collagen and von Willebrand factor [69]. However, platelet activation can also be initiated by other molecules such as serotonin [70], collagen [71], and adrenaline [72]. 

There are three main methods of PRP activation: calcium chloride activation, thrombin activation, as well as the use of exogenous energy sources (ultrasound, laser) [73].

### 4.1. Activation by Calcium Chloride

Calcium chloride is regarded as one of the safest and most cost-effective PRP activators, with no documented adverse effects, in contrast to bovine (xenogeneic) activators. This agent induces a rapid coagulation reaction in PRP, resulting in platelet degranulation and the release of growth factors. This method is particularly suited to clinical practice where rapid and uncomplicated activation is required [74].

Sadeghi-Ataabadi et al. compared different concentrations of calcium chloride (2.5, 5 and 10%) for PRP activation. The properties of the fibrin matrix and its ability to induce fibroblast proliferation were evaluated. Higher rates of cell adhesion and cell proliferation were obtained when 2.5% calcium chloride was used. In cultures with PRP activated with 10% calcium chloride, cells with the spindle-shaped morphology and parallel configuration of stress fibers were observed, whereas in cultures with PRP activated with lower concentrations of calcium chloride, typical fibroblast cells and stress fibers distributed in a reticulate configuration were observed [75].

Also, in a study by Hamilton et al., research was conducted to investigate the effect of calcium chloride activating agent on the concentration of growth factors depending on different states of physical activity. A significant increase in the concentration of the growth factors IGF-1 and PDGF-AB was confirmed, as well as a decrease in the concentration of HGF [76].

### 4.2. Activation by Thrombin

Bovine thrombin has been associated with adverse reactions, including bleeding, thrombosis, and an immune reaction. The use of autologous thrombin can effectively eliminate the risk of an immune response against PRP activated by this method. [77]. Autologous thrombin can be obtained from serum samples or using calcium gluconate for PRP coagulation [78]. Thrombin is the strongest agonist of platelets and is also responsible for converting fibrinogen into fibrin to stabilize clots [79]. The activation of platelets by thrombin is mediated by protease-activated receptors (PARs) [80]. Thrombin-induced activation is dose-dependent, with PAR-1 mediating platelet activation at low thrombin concentrations and PAR-4 at high thrombin concentrations [81]. 

In a study by Huber et al., it was observed that the activation of PRP using different concentrations of autologous thrombin did not promote a higher release of growth factors. Thus, the researchers concluded that the activation of PRP with autologous thrombin is not necessary when PRP is used in suspension form [78].

### 4.3. Activation by Exogenous Sources of Energy

Exogenous source activation methods utilize mechanical (ultrasound waves) or thermal energy (laser source) to stimulate platelet degranulation. In a study by Wu et al., it was observed that PRP activated with ultrasonic waves generated by piezoelectric ceramics can provide higher platelet concentration and less erythrocyte contamination than a commercial centrifugal device [82]. In a study by Farid et al., the combined application of the laser activation of stromal vascular fraction (SVF) and PRP for multiple sclerosis therapy was experimented with [83]. These methods allow for the precise control of the PRP activation process. However, the equipment and expertise required for ultrasound or laser activation may make these methods less accessible.

### 4.4. A Comparative Analysis Between Activated and Non-Activated PRP

In a study by Vahabi et al., the effects of activated and non-activated PRP at different concentrations on the proliferation of HGF fibroblasts and MG63 51 osteoblasts were compared. The results revealed that 10% activated PRP had the greatest effect on HGF proliferation after 24, 48, and 72 h. When the concentration was increased to 25, 50, and 75% in the activated PRP groups, the proliferation rate decreased. Changes in the proliferative activity of fibroblasts were associated with changes in pH after changes in PRP concentration [84]. The MG63 cell line showed the highest proliferative potential in the presence of 25% activated PRP, 10% activated PRP, and 25% inactivated PRP after 24, 48, and 72 h, respectively. However, it was noted that no significant differences were observed with the FBS-full DMEM group in vitro [85]. 

In the Simental-Mendía study, a systematic review and meta-analysis comparing the clinical efficacy of activated and inactivated PRP in the treatment of knee osteoarthritis (OA) determined that exogenous PRP activation revealed significant relief of pain and functional status compared to inactivated PRP. Notably, in the described studies in which inactivated PRP was used for therapy, no significant improvement in the clinical status of the patients was reported either in terms of pain or functional status compared to placebo. In this systematic review, seven of the nine studies that included exogenous platelet activation used calcium chloride and calcium gluconate, the latter showing the best results in this meta-analysis [86].

Gentile et al. evaluated inactivated and activated PRP for the therapy of androgenic alopecia, a common form of hair loss that affects up to 50% of men by age 50 and nearly 50% of women during their lifetime [87]. As a result, it was determined that, in fact, patients who received inactivated PRP had a greater increase in hair count and overall hair density than patients who received activated PRP [87]. The greater improvement in hair growth parameters with inactivated PRP compared with activated PRP may reflect the greater efficiency of the in vivo role of thrombin in platelet activation and in the distribution of activated platelet contents in the body compared with in vitro calcium activation and injection. Moreover, the administration of inactivated PRP may promote the production of thromboxane A2 by platelets after their in vivo activation by inducing circulating platelets and enhancing their aggregation [88].

Lee et al. found differences in determining the levels of growth factors PDGF and TGF-β in activated PRP, inactivated PRP, and whole blood compared to the data of Weibrich et al. [7]. Such differences in growth factor quantification appear to be multifactorial and include variability in the amount of proteins contained in platelets in different patients, different degrees of platelet concentration during PRP preparation, activation, or inactivation, and different degrees of platelet membrane rupture and degrees of platelet activation at the time of measurement. For example, in a study by Xiong et al., age and gender were shown to affect PRP composition in healthy patients [89]. As a result, it was determined that PRP from male patients contained higher levels of cytokines such as IL-1β, insulin regulated aminopeptidase, TNF-α, and growth factors such as PDGF-BB, VEGF, and TGF-β1 compared to female patients. Age differences were less correlated with growth factor levels. PRP from older patients had a lower IGF-1 content. Proteomic variability was observed within experimental groups. These data support a personalized approach to PRP therapy and highlight the need for a better understanding of the relationship between the level of expressed growth factors in PRP and clinical outcomes [89]. 

Activated platelets exhibit significant changes related to cytoskeletal elements, microtubule flexibility, and the centralization of βII-spectrin and vimentin. The expression levels of both α-granules and dense granules increased until the intermediate stage of the activation process but gradually decreased at a later stage. In addition, activation caused platelet granules to fuse with the open tubule system, releasing contents to attract additional platelets [90]. Thus, activation is a crucial step that can affect the number of bioactive molecules released and, consequently, the healing of damaged tissues. However, in some cases in clinical practice, some physicians prefer to administer inactivated PRP relying on spontaneous platelet activation occurring after exposure to native collagen present in connective tissues [91].

## 5. Application of PRP in Anti-Aging Clinical Trials

### 5.1. Treatment of Tendons, Ligaments, and Muscles

The elasticity of muscle ligaments and tendons decreases with age due to insufficient collagen and elastin production [92]; therefore, many diseases related to these structures are associated with age-related changes. Stimulating regeneration processes, PRP therapy will be effective in the treatment of connective tissue structures (ligaments, tendons) and muscles [93]. It can play a role in both acute and chronic conditions, and the desired results of PRP therapy include several benefits, including reducing pain and physical discomfort and stimulating tissue regeneration.

The most studied aspects in the field of PRP therapy are the methods of treatment of elbow tendons (lateral and medial epicondylitis), the Achilles tendon, and rotator cuff tendons of the shoulder. The use of PRP can help accelerate the recovery of tendons, improve the condition of elderly patients, and provide both short- and long-term relief for injuries to tendons and ligaments.

Lateral epicondylitis is an inflammatory process of the elbow region, in the place where the muscles attach to the external epicondyle of the shoulder. In traumatology and orthopedics, it is a widespread pathology, more often detected in individuals aged 30–50 years. People who, due to household or professional duties, often have to perform intensive repetitive movements of the hands are mostly ill. PRP injections for elbow diseases, especially lateral epicondylitis, have been investigated in numerous randomized controlled trials and systematic reviews, demonstrating different levels of evidence [94]. According to the results of a meta-analysis performed by Chen X, PRP can help reduce pain and improve function in lateral epicondylitis and shoulder injuries. In 2020, a systematic review and meta-analysis by Chou et al. confirmed the effectiveness of PRP as a primary or secondary treatment for lateral epicondylitis [95].

In a study involving patients with lateral epicondylitis in the PRP-treated group, gradual improvement was observed over the course of a year. At the same time, in the group where corticosteroid therapy was used, the condition worsened [96].

Achilles tendon injuries are also common and can occur with active foot movements. Many clinical studies on the effect of PRP on the restoration of Achilles tendon function have not shown positive results [32,97]. One recent placebo-controlled, multicenter, randomized controlled trial with two parallel groups with blinded participants and outcome assessment once again proved that PRP injection did not improve patient function or quality of life two years after an acute Achilles tendon rupture compared to placebo. The data from this study indicate that PRP does not benefit patients with Achilles tendon rupture in the long term [98].

### 5.2. Osteoarthritis

Osteoarthritis is a heterogeneous disease affecting all synovial joints (hand, knee, spine). Aging is considered one of the most significant risk factors for the development of OA. Studies indicate the presence of various cellular mechanisms through which aging contributes to the progression of degenerative processes in the joints in this disease [99].

Joint replacement is the most common treatment for severe OA. For mild to moderate OA, other forms of therapy are used, including PRP injections. PRP injections can provide short-term symptomatic relief and functional improvement. However, the clinical use of PRP in osteoarthritis therapy is controversial due to conflicting results and methodological limitations noted in the existing scientific literature.

Several meta-analyses and systematic reviews have reported a positive effect of PRP injections on reducing pain and improving function in knee OA compared to alternative treatments such as the use of hyaluronic acid (HA) and saline solution [100,101,102]. However, these studies are labeled with a Level II evidence rating and limit the validity of the results [92]. In addition, the optimal PRP preparation, concentration, and injection protocol were not standardized, contributing to the variability of the study results [101].

The combination of PRP and GC has been studied in practice, but the results of clinical trials have yielded mixed results. In a study of hip OA, PRP injections, as well as HA injections, provided significant clinical improvement at months 2 and 6, but in combination with HA, no additional benefits were observed over a 12-month period compared with HA alone [103]. In contrast, another study reported that the PRP-HA combination provided a longer-lasting effect and better outcome than PRP or HA alone [78]. Discrepancies between these studies may be due to differences in the concentration of the mixture.

However, recent studies show that low-white-blood-cell PRP injection has positive long-term effects when compared to glucocorticosteroids and HAs [104]. In contrast, another randomized controlled trial found no difference between GC and LP-PRP on pain criteria, but there were significant improvements in other patient-reported measures, with results favoring PRP over HA. There was also a trend toward lower levels of pro-inflammatory cytokines in patients in the PRP group, suggesting that the anti-inflammatory properties of PRP may contribute to symptom improvement (NCT02588872) [105].

Microfragmented adipose tissue therapy (MFAT), which has a heterogeneous cellular composition (including MSC), is also used to treat OA. However, comparative studies of MFAT are lacking, which is why the aim of the study by Baria M. et al. was to compare the patient-reported outcomes of a single injection of PRP and MFAT for knee OA. Both PRP and MFAT demonstrated clinically and statistically significant improvement in all assessment parameters in patients with knee OA 12 months after the injection. Despite this, data analysis revealed no statistically significant differences between the groups, suggesting a similar efficacy of the two treatments. These results may have important implications for clinical practice, as they indicate that it is possible to choose between PRP and MFAT, depending on individual preferences and patient characteristics, without the risk of worsening the clinical outcome (NCT04351087) [106].

All these studies support the controversial value of PRP injections for the treatment of OA, which means that new research is needed in this area of PRP injection use.

### 5.3. Periorbital Dark Circles and Wrinkles

The use of PRP injections, not only for medical but also cosmetic purposes, has been gaining popularity in recent years. Age-related anatomical changes in the tissues of the middle part of the face, such as the atrophy of subcutaneous fat and soft tissue degeneration in the cheekbone area, increase the darkness around the eyes [107]. Hormonal changes, exposure to ultraviolet rays, and taking certain medications, including oral contraceptives, can also affect the appearance of wrinkles and dark circles under the eyes [108].

In a recent study, Roohaninasab M. et al. compared the efficacy and safety of SVF, PRP, and neodymium laser (Nd: YAG laser) with nanofat injections in reducing dark circles and wrinkles under the eyes. The authors concluded that the combination of procedures was more effective in reducing dark circles under the eyes [109].

PRP is also used for facial rejuvenation, photoaging, and periocular wrinkles. In a single-center, double-blind, randomized controlled trial, Ya-Wen Tsai et al. compared the efficacy of PRP and platelet-poor plasma (PPP) for facial rejuvenation. Both methods showed significant improvement on global esthetic perception scales and the modified Fitzpatrick wrinkle scale for periocular wrinkles, with no significant difference between each. However, despite the positive changes in scores, improvements in the scales assessing wrinkle severity for nasolabial folds were not observed in any of the groups receiving PRP or PPP therapy. This indicates the possible limitations of the efficacy of these techniques on specific areas of the face, such as the nasolabial folds, and emphasizes the need for further research to better understand their effects on different wrinkle types and skin areas [110].

Overall, the findings may serve as a basis for developing more targeted and individualized approaches in esthetic medicine, allowing physicians to more effectively address patients’ problems, considering the specific anatomy and skin condition. Further research in this area will help to optimize treatments and improve outcomes, which in turn will improve patient satisfaction and quality of life.

### 5.4. Erectile Dysfunction

Erectile dysfunction (ED) is a common type of sexual dysfunction in men that is characterized by difficulty in achieving or maintaining an erection. This condition can manifest itself in a variety of ways, including the inability to achieve an erection.

According to studies, the prevalence of erectile dysfunction increases with increasing age, suggesting that older men are more likely to experience the problem [111].

The clinical study by Dr. Ranjith Ramasamy (NCT04396795) evaluates the efficacy and safety of PRP in the treatment of ED [112]. This study was initiated to investigate the potential of PRP as an alternative treatment option for men suffering from this common problem. Participants underwent a pre-treatment examination, including an assessment of the degree of ED using the International Erectile Function Scale (IIEF). During the study, participants received PRP injections in the penile area. At the end of the treatment, participants were re-evaluated to determine changes in erectile function. The study confirms the potential of PRP as an effective treatment for erectile dysfunction, opening new possibilities for men suffering from this problem. Nevertheless, further studies involving larger samples and long-term follow-up are needed to establish the sustainability of the results and to determine the optimal treatment parameters for final conclusions.

The evaluation of the safety and efficacy of PRP injections as a therapeutic agent for mild to moderate erectile dysfunction was also investigated in two other clinical trials [113,114]. In both studies, the PRP group showed significant improvements.

A randomized, double-blind, placebo-controlled trial conducted a comprehensive evaluation of the efficacy and tolerability of PRP injections in individuals with mild to moderate ED. The study revealed a substantial enhancement in erectile function within the PRP-treated cohort, with a remarkable 69% improvement when compared to the placebo group. Throughout the study period, no adverse events were reported (NCT04050020). The PRP injection therapy described in the study appears to be a safe and efficient short-term solution for mild to moderate ED [114].

Nevertheless, in the second study, the PRP group demonstrated a substantial improvement compared to the placebo group at 1 and 3 months after the intervention, although this improvement was slightly diminished at 6 months [113]. The findings of the research showed that the application of PRP led to a substantial enhancement in erectile function for the majority of the participants. The participants expressed their overall contentment with their sexual life. Furthermore, the study did not record any severe adverse effects, which suggests the safety of the procedure. PRP is a secure and promising approach for treating mild to moderate erectile dysfunction.

### 5.5. Healing of Wounds and Scars

The number of esthetic surgery procedures correlates with the age of patients. According to statistics, the majority of plastic surgery patients, published on the website of the American Society of Plastic Surgeons, plasticsurgery.org, were aged 40–54. That is why postoperative scars on the face and body after plastic and reconstructive surgeries are a visible esthetic problem that can seriously affect the quality of life, including patients of the older age group. Studying the effect of PRP on postoperative scar revision is also a relevant area of research. In a recent randomized study, Menchisheva Y. et al. evaluated the results of PRP injection during surgery. The injection was given subcutaneously during surgery after wound closure. As a result, the change in scar width was half as pronounced in the PRP group, indicating a positive therapeutic effect [115].

Hersant et al. investigated the effect of PRP injections on keloid scars and reported complete remission after 2 years of follow-up in 53% of keloids, but the number of recurrences reached 29% [116]. A further study was conducted to evaluate the efficacy of PRP application in breast reduction surgery and abdominoplasty. The study documented a favorable impact of PRP on the reduction in complications following breast reduction surgery, with no instances observed in the experimental group and 25% in the control group with a fixed hematoma. In patients undergoing an abdominoplasty procedure, the incidence of complications was 37.5% in the control group and 12.5% in the group that received PRP. Nevertheless, the same study demonstrated that there were no statistically significant differences in the healing of postoperative scars between the control group and the experimental group after a 12-month follow-up period [117]. 

### 5.6. Alopecia Areata

Androgenetic alopecia is a pathological state characterized by the shedding of hair on the scalp in a male-patterned manner, resulting in the atrophy of hair follicles under the influence of androgens. The prevalence of this condition is closely linked to the age of the affected individual [118].

PRPs are among the newer treatments for androgenic alopecia. Behrangi E. et al. aimed to investigate the effect of adding SVF to PRP and compared it with PRP injection alone. Initially, 20–30 mL of fat was taken from the lower abdomen of patients, and SVF was isolated using 0.1% collagenase. PRP was then isolated by centrifugation. The injections were performed in three stages; the PRP group was injected with PRP at all 3 visits, 1 month apart, and the SVF-PRP group was injected with SVF at the first visit and PRP at the two subsequent visits. In summary, adding one SVF injection to two PRP treatment sessions compared with three PRP injections alone made no significant difference. But the authors suggest that more SVF injections are needed for a statistical difference. No serious complications were reported during the treatment and follow-up of the patients [119].

In a randomized trial in men, PRP injections had a higher incidence of side effects than FDA-registered minoxidil: 53% PRP, 37% minoxidil. One participant developed contact allergic dermatitis to the product after one week of use, manifested by erythema and a burning sensation over the hair growth line. In summary, the authors concluded that PRP is effective in treating moderate-to-severe androgenic alopecia in men, although perhaps not unlike minoxidil. Precise results could not be recorded because of the short duration of follow-up [120] (Table 2).

## 6. A Discussion of Perspectives on the Application of the PRP

To date, the role of PRP in modern medicine is ambiguous. On the one hand, PRP products do accelerate regenerative processes and do not cause serious side effects. On the other hand, a number of studies that we cited in this article indicate a lack of efficacy. We assume that the future of PRP therapy will be closely related to different variants of combination therapy [121,122]. However, more clinical trials are needed to establish the efficacy of such approaches. In addition to regenerative and esthetic medicine, where PRP is currently active, this approach has also been tested in other related fields. There are several studies showing the favorable effect of PRP in the role of adjuvant therapy in the treatment of bladder cancer [123,124]. However, the role of PRP in the treatment of oncology is highly controversial, as discussed in a number of papers [125]. For example, the presence of growth factors can lead to angiogenesis in vitro [126]. Another study evaluated the efficacy and safety of PRP after breast tumor removal in patients with early-stage breast tumors. There were no complications or recurrences at 30 months of follow-up [127]. The question of the need for and effectiveness of PRPs remains open at this time and needs additional in vitro and in vivo studies. 

## 7. Conclusions

PRP-based therapy is an interesting solution as a personalized therapeutic approach. Thanks to the natural functions of platelets, PRP can indeed have a beneficial effect on tissue regeneration, scar healing, esthetic problems, and the treatment of musculoskeletal disorders. The existing variety of PRP modifications and the added data on molecular mechanisms of action will allow this field to rapidly gain momentum over the next few years. However, based on the analysis of clinical trials, it can be concluded that the use of PRP is not always accompanied by positive treatment results. As a result of the analysis of clinical trials, we can conclude that the activation process is not used in most cases. Despite the rare side effects, it is quite common to find a lack of efficacy in studies, which may be due to problems of unsuitable sample preparation, the incorrect selection of an activating agent, or the lack of activation in general. Further studies will help to establish in more detail the mechanisms of PRP products in diseases associated with aging, thereby improving the efficacy of this procedure.

## Figures and Tables

**Table 1:** Commercial FDA-approved PRP isolation kits and systems.

Name of Commercial Kit or System	Mode	Cost
ACP-system (Arthrex, Munich, Germany)	Double-Syringe System	≈124$
System Angel cPRP (Arthrex, Munich, Germany)	Patented platelets sensor system, precision separation using three-sensor technology fully automated process, separation of blood into three fractions	≈16,900$
Magellan Autologous Platelet Separator; (Arteriocyte Medical Systems, Hopkinton, MA, USA)	Fully automated patient-to-patient process, concentrating PRP	≈11,900$
DrPRP-Kit^®^ (REMI Laboratory Instruments, Seoul, Republic of Korea)	Patented design shape, clear separation of PRP, high concentration	N\A

**Table 2:** Clinical outcomes of age-related diseases.

Disease Type	Type of PRP Product	Activation	PRP Production Method	Type of Research	Results (Comparison with Control Group)	Research Number
Lateral epicondylitis	Autologous PRP	-	Centrifugation	Randomized, controlled parallel study, level of evidence of 1	After 12 months, VAS was 81.4% lower and DASH was 21% lower	[96]
Achilles tendon rupture	L-PRP	-		Randomized, multicentre, parallel blinded, level of evidence of 1	No evidence of efficacy has been identified	[98]
Osteoarthritis	Autologous PRP	-	Two stages of centrifugation	Randomized controlled trial, level of evidence of 1	After 12 months, the VAS score was 47.7% lower	[103]
Osteoarthritis	LP-PRP	-	ACP-system (Arthrex, Munich, Germany)	Double-blind placebo-controlled single-center study	Changes in pain levels during the first 6 months compared to baseline were small (within ±5 NRS scores)	[104]
Osteoarthritis	LP-PRP	-	ACP-system (Arthrex, Munich, Germany)	Randomized controlled trial, level of evidence of 1.	There were no significant differences on the WOMAC score	[105]
Osteoarthritis	Autologous PRP	Ultrasound	Angel cPRP system using 2% haematocrit; Arthrex	Randomized controlled trial; level of evidence of 2.	A 40% reduction in VAS score from day 0 over 6 months was observed	[106]
Periocular wrinkles	PRP и PPP	-	Two stages of centrifugation	Single-center double-blind randomized controlled trial	Mean GAIS PRP scores were 3/1 ± 0.5, PPP scores were 2.9 ± 0.5	[110]
Erectile dysfunction	Autologous PRP	-	Two stages of centrifugation	Placebo-controlled study	Improvement in 70% of subjects 6 months after injection (54% more than in the control group)	[114]
Erectile dysfunction	PRP	-	Magellan Autologous Platelet Separator; Arteriocyte Medical Systems, Hopkinton, MA	A double-blind randomized placebo-controlled trial, evidence level of 1.	After 6 months, the IIEF score was 42% higher than in the control group	[113]
Keloid scars	PRP	-	Plasma obtained from the commercial RegenKit^®^-BCT kit	Prospective study	Complete remission after 2 years of follow-up in 53% of keloids, but the number of recurrences reached 29%	[116]
Androgenetic alopecia	Autologous PRP, SVF-PRP	-	Two stages of centrifugation	Randomised	PRP: hair count increased by 42.7% SVF-PRP: increase by 29.3%	[119]

PRP—platelet-rich plasma, L-PRP—leukocyte and platelet-rich plasma, LR-PRP—leukocyte-rich PRP, LP-PRP—leukocyte-poor PRP, HA—hyaluronic acid, PPP—platelet-poor plasma, SVF—stromal vascular fraction.

## Data Availability

Not Applicable.
